# Extensive Traumatic Ulceration—Case 1

**Published:** 1899-12

**Authors:** T. J. Biggs

**Affiliations:** New York


### EXTENSIVE TRAUMATIC ULCERATION—CASE I.

By Dr. T. J. Biggs, New York.

F. L—— came to the Demilt Dispensary, December 22d, 1892, having sustained, two weeks previously, while at work as brakeman on the Long Island Railroad, a large laceration of the forearm, which was denuded of the integument and superficial and deep fasciæ, to the extent of seven inches in its long axis and three and a half inches in width. For two weeks he had been treated at several dispensaries and hospitals, under antiseptic methods, without preventing the formation of a large-sized black slough, about two inches in length and extending down to the periosteum. Furthermore, as a result of the severe blow which the muscles of the region had sustained, total paralysis of the forearm existed. He had been advised to have the arm amputated. This he refused to submit to; hence his appearance at the Demilt Dispensary for treatment under Dr. Biggs.

The preliminary treatment here pursued was the employment of such applications as would tend to hasten the removal of the slough and stimulate the wound to a condition of healthy granulation—namely, balsam of Peru, peroxide of hydrogen, etc.

The desired condition was obtained in about ten days' time, when it was decided to undertake to heal the wound by skin-grafting. The method employed, however, was not that of Thiersch, but the direct application of small graft points at intervals to be nourished by topical blood supply.

After careful cleansing the surface of the wound with Thiersch's solution (Corrosive Sublimate was not used, as it is believed to coagulate the albumen in the tissue and interfere with the adhesion of the graft; and the sovereign depurator, Peroxide-on-bovinine, was not yet known) and preparing a clean area on the other arm, from which the grafts were to be taken, several small pieces of skin were snipped off from this surface, washed with the Thiersch solution, and carefully placed over the surface of the wound at a distance of one inch apart each. Eight of these minute grafts were employed in all. Directly over the grafts were placed strips of rubber tissue, soaked in Thiersch solution, and over this, sterilized gauze wet with the same solution. The dressing was completed by the application of more rubber tissue over the whole forearm, and finally a splint and an evenly-applied bandage.

On inspection after forty-eight hours, the grafts were barely adherent, and had lost their pinkish hue, showing a want of nutrition from the unaided natural circulation. One had been brushed off, leaving only seven, scattered over the surface of the ulceration as sources of skin propagation. The dressing of bovine blood was now applied, and a peculiar effect was immediately experienced. Up to this moment, the patient had suffered incessant pain in the wound, so severe that, for two weeks and more previously, sleep could be obtained only by the use of powerful anodynes. The application of bovinine was instantly followed by a sense of most grateful relief, and within a few minutes the pain had wholly disappeared, and (the bovinine dressing being continued) never returned!

The grafts were afforded constant artificial nutrition by bovinine, on which they thrived, developed and became permanently adherent. The dressings from now on were changed about every forty-eight hours until at the end of about eight weeks, when the wound was found to be absolutely and entirely well, having become covered with healthy epithelium, which had traversed the surface of the wound, having been developed alike from the circumference and from the several grafts. March 6th, 1894, the patient was discharged in perfect repair; the wound being fully covered with healthy skin, having but a comparatively small pink cicatrix, which was not sensitive, hard, or contracting the surrounding tissues.

It remains only to add, that the arm, which had been from the time of the injury totally paralyzed, was restored to full power.

This brief, painless, and uninterrupted cure, occupying only sixty-four days, and requiring only seven minute points of graft skin for some twenty-five square inches of new growth which the nourishing blood-preserve built steadily out from each graft over the bare intermediate spaces—created an epoch in tissue construction of which the promise is even yet immeasurable: remembering as we should, that in principle and substance it was but a repetition of a clear physiological process which the blood-preserve had already made invariably practicable in hundreds of cases on record, and which has since been incredibly extended in surgical practice with supplied blood.

